# Supplementary material for: Women empowerment through involvement in community-based health and nutrition interventions: Evidence from a qualitative study in India
Source: PLoS One. 2023 Apr 20;18(4):e0284521. doi: 10.1371/journal.pone.0284521 (PMC10118087; doi:10.1371/journal.pone.0284521)
Supplement: Attachment — Submitted filename: R1_Response to reviewer and editor comments_24032023.docx [file pone.0284521.s001.docx]

**Response to review and editor comments**

| **Comments** | **Response** |
| --- | --- |
| Journal requirements:  When submitting your revision, we need you to address these additional requirements.  1. Please ensure that your manuscript meets PLOS ONE's style requirements, including those for file naming. The PLOS ONE style templates can be found at  <https://journals.plos.org/plosone/s/file?id=wjVg/PLOSOne_formatting_sample_main_body.pdf> and [https://journals.plos.org/plosone/s/file?id=ba62/PLOSOne_formatting_sample_title_authors_affiliations.pdf](https://secure-web.cisco.com/15DC0o2kfT75zEPATyvIlvVaCbOg-l9lUic5BECDRQUTXeNuIu10a_ndcIx0LvHh9xlItiw6SmzTsqZ62gHW8N2Ss_EFJhhUZjfJX0Rp-znR09W4yhB5mGUL1fIsJE5zBt0fiDT9TaWNiosxTATpuEWynKFJFLZh2kKoeHt3brAUmIkz7MRJHhCdylR1nslEZZe7vWafGpnswlUvK3O1T53zDHDqsI9jm-aoq1QUEHiBs4udqBaQhYXj9mwtmf7RlopmTG3QwKSCJbgKHWSPfB6UgGXIY4pSUGCw8j1rEsShyGhypUk1ScOw8W00xvB5_6WCKpCaX7mmOLQNizZ1tT_yQo9z4PXQ7Vuc0ob4Yh5cNDmJZ9WgFBGLgKaSXRIOZ7mf670FQ9g3WmCGzeodxWrCYqPkQQv3E4sFu98QJ04OLILCiNruOyOMaSdq6zCVS4FiXpAGtiXHmFluXlVWXsQ/https%3A%2F%2Fjournals.plos.org%2Fplosone%2Fs%2Ffile%3Fid%3Dba62%2FPLOSOne_formatting_sample_title_authors_affiliations.pdf). | The revised manuscript follows the suggested style requirements |
| 2. Please provide additional details regarding participant consent. In the ethics statement in the Methods and online submission information, please ensure that you have specified what type you obtained (for instance, written or verbal, and if verbal, how it was documented and witnessed). If your study included minors, state whether you obtained consent from parents or guardians. If the need for consent was waived by the ethics committee, please include this information. | The method section includes additional details regarding type of participant consent. |
| 3. We note that the grant information you provided in the ‘Funding Information’ and ‘Financial Disclosure’ sections do not match.  When you resubmit, please ensure that you provide the correct grant numbers for the awards you received for your study in the ‘Funding Information’ section. | The research received grants under Programme Code & Title- Nutrition 200-Swabhimaan; Project Code & Title: 200-203-03-Swabhimaan, from UNICEF, New Delhi, India.  The aforementioned grant number is provided in the Funding information Section in the cover letter and online financial disclosure section. |
| 4. In your Data Availability statement, you have not specified where the minimal data set underlying the results described in your manuscript can be found. PLOS defines a study's minimal data set as the underlying data used to reach the conclusions drawn in the manuscript and any additional data required to replicate the reported study findings in their entirety. All PLOS journals require that the minimal data set be made fully available. For more information about our data policy, please see <http://journals.plos.org/plosone/s/data-availability>.  Upon re-submitting your revised manuscript, please upload your study’s minimal underlying data set as either Supporting Information files or to a stable, public repository and include the relevant URLs, DOIs, or accession numbers within your revised cover letter. For a list of acceptable repositories, please see <http://journals.plos.org/plosone/s/data-availability#loc-recommended-repositories>. Any potentially identifying patient information must be fully anonymized.  Important: If there are ethical or legal restrictions to sharing your data publicly, please explain these restrictions in detail. Please see our guidelines for more information on what we consider unacceptable restrictions to publicly sharing data: <http://journals.plos.org/plosone/s/data-availability#loc-unacceptable-data-access-restrictions>. Note that it is not acceptable for the authors to be the sole named individuals responsible for ensuring data access.  We will update your Data Availability statement to reflect the information you provide in your cover letter. | The revised manuscript contains the following data availability statements.  “Data cannot be shared publicly because of ethical issues. Data are available from the Swabhimaan program at IIPS, Mumbai (contact: [iipsswabhimaan@iipsindia.ac.in](mailto:iipsswabhimaan@iipsindia.ac.in)) for researchers who meet the criteria for access to confidential data”. |
| Please review your reference list to ensure that it is complete and correct. If you have cited papers that have been retracted, please include the rationale for doing so in the manuscript text, or remove these references and replace them with relevant current references. Any changes to the reference list should be mentioned in the rebuttal letter that accompanies your revised manuscript. If you need to cite a retracted article, indicate the article’s retracted status in the References list and also include a citation and full reference for the retraction notice. | The reference list has been checked and found to be complete and correct. |
| **Reviewer 1** |  |
| Reviewer #1: General comments: This is an important topic reflecting on community participation of women from SHGs and its impact on their empowerment.  The three themes derived from the data are presented well however, as this paper primarily focuses on empowerment of SHGs, the barriers at personal and professional front could have been probed more in terms of: 1. Challenges at the level of family: Family member’s positive or negative attitude towards their job (especially of the spouse and in-laws ), how it influences their work? and how do they overcome such challenges? Does the recognition and role within community, has empowered their decision making ability within their household? (As decision making ability within household is an important indicator of women empowerment). Insight on such aspects will shed more light on the crucial role of family in women empowerment. 2. Challenges at the organization level: - Factors like hierarchical support/conflicts/discrimination, issues related to delay in incentive dispersal (This is a common issue faced by HCWs) and what motivates them to continue their work despite such challenges, if any. | Thank you for reviewing the paper and the valuable comments to enhance its quality.  As appreciated, efforts were made to present the three emerging themes from the data.  Nevertheless, in view of the suggestions, we have made revised the manuscript highlighting the barriers at personal and professional front. |
| Some minor comments: |  |
| The data collection method has been presented well except for few points that needs modification or further information needs to be added to make it more streamlined with qualitative study guidelines: | As suggested, we have done required changes to make data collection method more streamlined |
| 1. Line 154-156: Coding of data requires two or more independent researchers ensuring Intercoder Reliability (ICR), kindly provide the information on number of researchers involved in coding apart from the primary author. | Both the first and the corresponding author were involved in initial coding of 3 IDIs. The codes were discussed at length and a comprehensive code list was prepared in consensus, to ensure ICR.  Specifically, the following sentences are added in the revised manuscript.  “The coding process started with identification and coding of three IDIs, independently by the first and the corresponding author. To ensure, inter-coder reliability, both the authors discussed the initial emerging codes and finalized the code list. Subsequently, the first author coded the data and briefed the study team about coding categories and the emerging themes from time to time.” |
| 2. The number of respondents who were approached, how many refused to participate and reason for refusal. | We could interview all the chosen respondents. Specifically, the following sentences are added in the revised manuscript.  “We approached 25 PSs and could interview all of them at their convenient time and place. There was no refusal as they were active partners in the implementation program. Specifically, the present study is based on 25 IDIs of PSs from three Indian States-Bihar (n=9), Odisha (n=8), and Chhattisgarh (n=8)”. |
| 3. Was the transcript shared with the participants for comments or correction? | Individual respondents were briefed on the points raised/told by them to avoid any misunderstanding/assessment by the interviewer. However, the transcripts which were prepared after the interviews were not shared with them. |
| 4. Duration of IDIs | The duration of IDIs ranges between 45- 65 minutes. |
| Line 94-95: “This paper attempts to examine the impact on the implementors and their role in implementing the intervention in enhancing empowerment”: This line could be modified as it confuses whose empowerment authors are referring to, SHGs or community? Since, the participants are only PSs (Poshan Sakhis) it would be better to put the rationale in their terms. Suggestion: “ This paper explores the role of SHGs in improving the effectiveness of community health interventions and its impact on their self-empowerment. | Appreciate the suggestion. We have modified the sentence as suggested. |
| Line 248: “registration, enhanced antenatal care (ANC), consumption of IFA tablets/syrup” Suggestion: Kindly mention consumption of nutrition supplements or mention the full range of supplements given i.e IFA, Calcium and Albendazole tablets. | As suggested, we have modified the sentence |
| Overall the paper provides a good insight on the role of PSs in community health intervention and how it is beneficial for their personal growth. If the authors can respond to the above queries, then this paper may be considered for publication. | Thank you for appreciating the contribution of this paper. We have incorporated all the comments of the reviewer in the revised manuscript to be considered for publication. |

Thank you
